# Childhood adversities and depressive symptoms in women: a population-based cross-sectional study*

**DOI:** 10.1590/1980-220X-REEUSP-2026-0138en

**Published:** 2026-09-28

**Authors:** Tiffani Matos Oliveira, Franciele Marabotti Costa Leite

**Affiliations:** 1 Universidade Federal do Espírito Santo, Programa de Pós-Graduação em Saúde Coletiva, Vitória, ES, Brazil.

**Keywords:** Adverse Childhood Experiences, Depression, Epidemiology, Women

## Abstract

**Objective:**

To investigate the association between exposure to adversities in childhood and the occurrence of depressive symptoms in women in adulthood.

**Method:**

Epidemiological, cross-sectional, population-based study with 1100 women from Vitória, Espírito Santo, in 2022. The International Childhood Adversity Questionnaire and the Beck Depression Inventory were used. In the analyses, crude and relative frequencies were calculated, with 95% confidence intervals. In the bivariate analyses, Pearson’s chi-square test was applied, and for the analyses of associations with adjustments for confounding variables, logistic regression was used. The analyses were performed using Stata 17.0.

**Results:**

Women with a childhood history of physical neglect (ORa: 1.59; 95% CI: 1.16–2.16), sexual abuse (ORa: 1.57; 95% CI: 1.03–2.39), and an incarcerated family member (ORa: 1.90; 95% CI: 1.18–3.05) were more likely to experience depressive symptoms in adulthood compared to those who did not have such experiences.

**Conclusion:**

The findings reveal how important and necessary it is to invest in intersectoral public policies for the protection of childhood and the tackling of economic inequalities, to minimize adverse events in this important phase of life, since they negatively impact health throughout life.

## INTRODUCTION

Adverse childhood experiences (ACEs) are a major public health problem and encompass a range of contextual conditions such as interpersonal losses, dysfunctional family environments, maltreatment (physical, psychological, sexual, and neglect), economic hardship, and community violence^(1,2)^.

International data indicates that millions of children are exposed to ACE^(3)^ annually, with significant prevalence rates found in countries in Europe and North America^(4)^. In the United States, specifically, data from 2022 revealed that more than 3 million children were subjected to abuse, and 1,990 deaths were recorded as being directly related to these experiences^(5)^.

In the Brazilian context, in the South and Southeast regions, the prevalence rates are equally alarming, with the occurrence of at least ACE ranging from 53.6% to 85%^(6,7,8)^. A study conducted in Pelotas, Rio Grande do Sul, identified that 85% of young people up to 18 years old have suffered at least one ACE, with a higher incidence among girls, who suffer four or more types^(9)^. Given this scenario, the literature highlights the importance of studies in the Brazilian context, considering the country’s socioeconomic particularities and the scarcity of data on this topic^(10)^.

Considering these particularities and the lack of population data on the subject, it becomes essential to understand how such experiences are incorporated by the female body. It is recognized that early experiences of adversity in childhood promote structural and functional changes in brain development, which can deregulate the endocrine and immune systems. These biological modifications increase susceptibility to a range of clinical conditions and psychological manifestations, such as some spectra of psychosis, anxiety, and depression^(9,11)^.

Specifically regarding depression, exposure to childhood maltreatment can increase the risk of developing depressive disorder in adulthood two to three times, with emotional abuse and neglect being strong predictors of this outcome^(12)^. This association is mediated by a complex interaction between biological (hypothalamic-pituitary-adrenal (HPA) axis, neural structure and function) and psychosocial (attachment and coping skills, stress, etc.) mechanisms; from one perspective, dysregulation of the HPA axis and alterations in neural structure and function occur; in contrast, insecure attachment patterns and compromised stress-coping skills are consolidated. By shaping emotional responses from early stages of development, these factors establish a vulnerability that persists throughout an individual’s life^(13,14)^.

The seriousness of this association becomes even more relevant due to the epidemiological scenario of the pathology, which affects almost 280 million people and is one of the most prevalent disorders and a leading cause of disability in the world^(15)^. In this context, the role of nurses in health services is of great importance, since these professionals often establish the first contact with users, facilitating the early identification of depressive symptoms and monitoring throughout the care process. The quality of the patient-nurse relationship can contribute to the expression of emotional complaints and adherence to proposed interventions^(16)^.

Given the above, understanding the relationship between childhood adversity and depression becomes imperative for guiding prevention and care strategies in the health field. This is so because despite the growing number of international studies on the relationship between childhood adversity and mental health outcomes, population-based research, especially focused on the female population, is still limited. Thus, this study aimed to investigate the association between childhood exposure to adversities and the occurrence of depressive symptoms in women in adulthood, in Vitória, Espírito Santo.

## METHOD

### Study Design

This is an epidemiological, observational, cross-sectional study, based on field research entitled “Violence against women in Vitória, Espírito Santo: A population-based study”, conducted in 2022.

### Local

The municipality of Vitória, Espírito Santo, had an estimated female population of approximately 163,853 in 2021, representing 49.98% of Vitória’s population, according to the Brazilian Institute of Geography and Statistics (IBGE).

### Population

The sample consisted of women aged 18 or older residing in the municipality of Vitória, Espírito Santo, who had or had had partners in the last 24 months.

### Selection Criteria

The inclusion criteria for the study established that participants had to be 18 years of age or older, who had had or currently have had an intimate partner in the 24 months prior to the interview. Women who lacked the ability to understand or communicate due to intellectual or sensory impairment and who, therefore, would not be able to answer the research questionnaire^(17)^ were excluded.

### Definition of the Sample

Sampling was carried out in multiple stages. Initially, the primary sampling unit was selected, which was the census tracts of the municipality of Vitória provided by the 2010 IBGE census. The total number of households in the urban area of Vitória, Espírito Santo, is 108,515, which was divided by 100 (the number of sectors to be visited) to obtain the systematic skip (1,085), respecting the probability proportional to the number of households and women within each sector. After selecting the census tracts, a list was made by socioeconomic level, and the number 513 (between 1 and 1,085) was randomly selected by the R statistical software, corresponding to the number belonging to the first defined tract. Finally, the remaining tracts (99) were selected, which occurred by summing the systematic skip from the initial sector (184) until the end of the list. Based on the selection of census tracts, households were then selected (randomly) according to the list available on the IBGE’s online platform. After the list of women eligible according to the study criteria was compiled, the women who were going to respond to the survey were randomly selected. To calculate the sample size, a confidence level of 95%, a power of 80%, and an exposed/unexposed ratio of 1:1 were taken into consideration. To this value were added 10% to account for losses, and 30% for confounding factors, and the sample size reached 1,100 women^(17)^.

### Data Collection

The research conducted a pilot study in December 2021, and data collection took place between January and May 2022, carried out by a team of properly trained interviewers, with field supervisors. Data collection was carried out in the women’s homes, using tablets and respecting their privacy and confidentiality^(17)^. The questionnaire was structured using REDCAP (Research Electronic Data Capture).

### Variables

The research used socioeconomic issues and family ­experi­ences as independent variables. Socioeconomic factors: Age range (18 to 34 years; 35 to 59 years; ≥60 years); Women’s education level (0 to 4 years; 5 to 8 years; 9 to 11 years); Race/color (white; black; brown and others); Family income (1st quintile – poorest; 2nd quintile; 3rd quintile; 4th quintile and 5th quintile – richest); Family experiences (Childhood adversities): Abuse (physical, sexual and emotional – no/yes); Neglect (physical and emotional – no/yes); Family conflicts (parental separation; incarcerated family member; family member with mental health problems; alcohol abuse by family members - no/yes).

The Childhood Adversity International Questionnaire (ACE-IQ) was used to assess ACE^(2)^, validated in Brazil^(18)^. The instrument aims to identify and assess past exposure to 13 different categories of ACEs, including sexual, emotional, and physical abuse, emotional and physical neglect, family violence, alcohol/drug use, mental illness or suicide within the home, family involvement in criminal activities, parental separation or divorce, among other forms, to evaluate the occurrence and frequency of adverse childhood experiences^(18)^.

The outcome variable under study, depressive symptomatology, was tracked using the Beck Depression Inventory (BDI) instrument validated in Brazil^(19)^. The BDI is a self-report measure composed of 21 items, where each item contains four statements. These individuals exhibit increasing degrees of intensity of depressive symptoms, with scores ranging from 0 to 3 on a Likert-type scale^(20)^.

The scale includes questions about: Sadness; Pessimism; Feeling of failure; Dissatisfaction; Guilt; Punishment; Self-loathing; Self-blame; Suicidal thoughts; Crying; Irritability; Social withdrawal; Indecisiveness; Change in self-image; Difficulty working; Insomnia; Fatigue; Loss of appetite; Weight loss; Somatic concerns; and Loss of libido. Thus, the scale is used as a way to screen for depressive symptoms in populations with or without a diagnosis of depression, aiming to identify the intensity of depressive symptoms^(20)^.

### Data Analysis and Processing

The statistical analysis began with an exploratory evaluation of the BDI scores. The verification of normality, performed using a histogram and confirmed by the Shapiro-Wilk test (*p* < 0.001) indicated asymmetry and non-normality of the data. Given this, the outcome variable was categorized based on Cunha’s cut-off points: 0 to 11 for minimal symptomatology; 12 to 19 for mild; 20 to 35 for moderate; and 36 to 63 for severe symptoms. The variable was then dichotomized, defining scores of 12 or higher as the presence of depressive symptomatology, while scores of 0 to 11 were classified as no depressive symptomatology^(19)^.

Data were analyzed using descriptive statistics, including crude and relative frequencies and their 95% confidence intervals (95% CI). For the bivariate analyses, Pearson’s chi-square test (χ^2^) was performed, considering a statistical significance of 5%. The association between the variables was determined using Logistic Regression and their respective confidence intervals (95% CI). For inclusion in the adjusted analysis, p-values <0.20 of the bivariate and the permanence in the adjustment when p < 0.05 were considered.

A hierarchical analytical variable entry model was adopted, grounded in the theoretical framework of the social determination of health^(21)^. The analytical model comprised two levels: the first (distal) encompassed childhood adversities (neglect, abuse, incarcerated family member, etc.), followed by the intermediate level, which comprised sociodemographic data (education, income), both directly impacting the outcome (Figure 1). The analyses were conducted using Stata statistical software, version 17.0.

**Figure 1:**
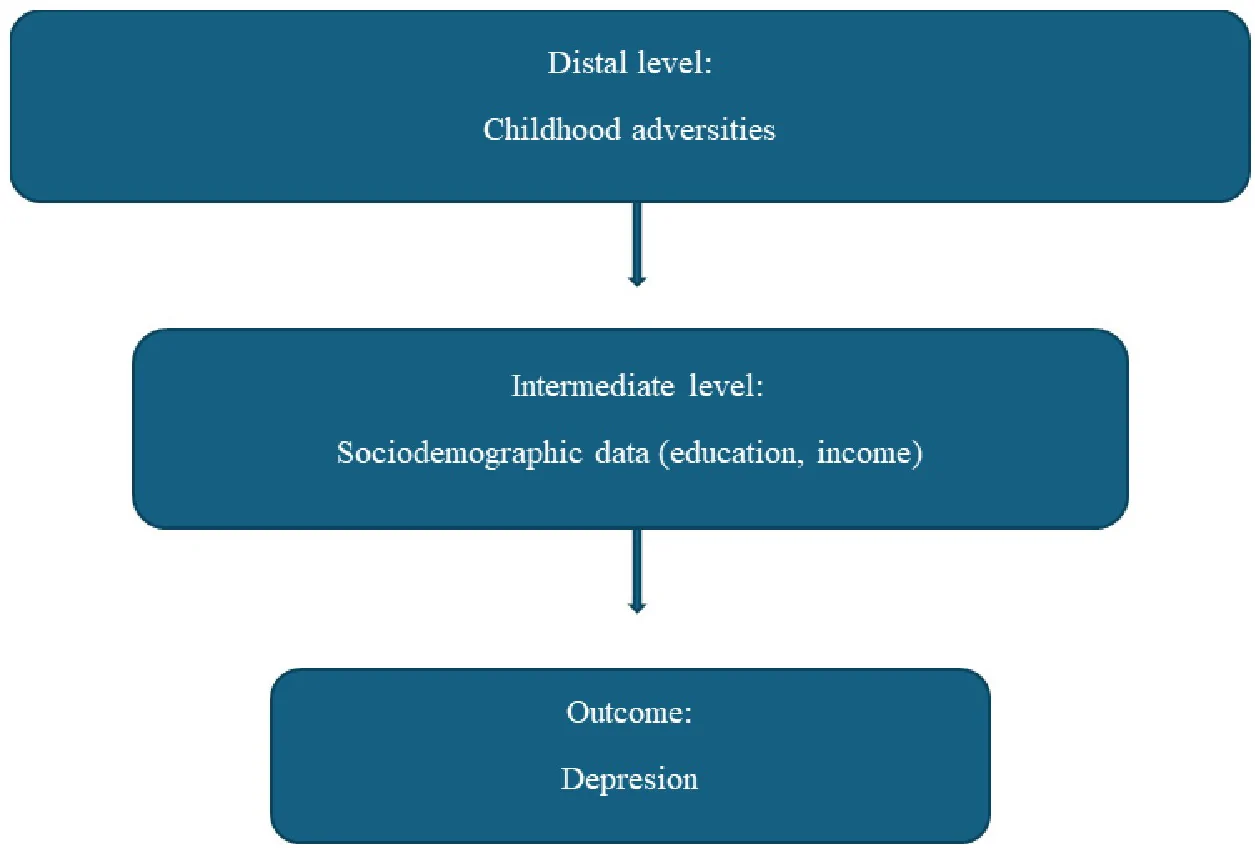
Sample analysis.

Source: prepared by the authors, 2025.

### Ethical Aspects

The project was submitted to the Research Ethics Committee of the Universidade Federal do Espírito Santo, and approved through opinion number 4,974,080, to comply with resolution no. 466/2012. All the women interviewed signed the Free and Informed Consent Form (FICF).

## RESULTS

A statistically significant relationship was observed between socioeconomic conditions and depressive symptomatology (p < 0.05). Women belonging to the lowest family income group presented a higher frequency of depressive symptoms (34.5%), as did those participants with fewer years of education (36.1%) (Table 1).

**Table 1:** Bivariate analysis between depressive symptomatology and sociodemographic and economic data among women residing in the municipality of Vitória, ES, Brazil, 2022.

	N (%)	95% CI	p-Value
**Age range**			0.221
18 to 34 years old	82 (27.6)	22.8–33.0	
35 to 59 years old	119 (22.6)	19.2–26.4	
≥60 years	59 (22.5)	17.9–27.8	
**Race-color**			0.419
White	102 (23.4)	19.5–27.6	
Black	54 (27.6)	21.7–34.2	
Brown and others	104 (22.9)	19.3–27.0	
**Women’s education**			0.033
0 to 4 years	22 (36.1)	25.1–48.8	
5 to 8 years	39 (27.5)	20.7–35.4	
9 to 11 years old	199 (22.5)	19.9–25.4	
**Family income**			<0.001
1st quintile (20% with the lowest income)	77 (34.5)	28.6–41.0	
2nd quintile	61 (28.5)	22.9–34.9	
3rd quintile	43 (18.7)	14.2–24.3	
4th quintile	48 (22.5)	17.4–28.6	
5th quintile (top 20% income earners)	175 (15.1)	10.8–20.6	

N: raw frequency and %: relative frequency.

95% CI: 95% confidence interval.

p-value Pearson’s chi-squared test.

The results of the bivariate analysis (Table 2) revealed that exposure to all categories of childhood adversities was related to the occurrence of depressive symptoms in adulthood (p < 0.05).

**Table 2:** Distribution of depressive symptoms among women residing in the Municipality of Vitória, ES, according to the types of adversities experienced in childhood – Vitória, ES, Brazil, 2022.

	N	%	95% CI	p-Value*
**Emotional Neglect**				<0.001
No	174	21.3	18.6–24.2	
Yes	86	32.1	
**Physical Neglect**				<0.001
No	139	19.0	16.3–22.0	
Yes	121	34.1	29.3–39.2	
**Sexual Abuse**				<0.001
No	210	21.9	19.4–24.6	
Yes	50	39.1	31.0–47.8	
**Physical Abuse**				<0.001
No	94	17.6	14.6–21.0	
Yes	166	30.1	26.4–34.1	
**Emotional Abuse**				<0.001
No	71	13.9	11.2–17.2	
Yes	189	32.9	29.1–36.8	
**Parental Separation**				0.006
No	118	20.6	17.4–24.1	
Yes	142	27.7	24.0–31.8	
**Incarcerated relative**				<0.001
No	219	22.0	19.5–24.7	
Yes	41	45.6	35.6–55.8	
**Family member with mental health problems**				0.001
No	193	21.8	19.2–24.7	
Yes	67	33.3	27.2–40.1	
**Alcohol abuse by family members**				<0.001
No	155	20.3	17.6–23.3	
Yes	105	32.5	27.6–37.8	

N: raw frequency.

%: relative frequency.

95% CI: 95% confidence interval.

p-value Pearson’s chi-squared test.

After adjusted analysis (Table 3), it is noted that belonging to the first quintile of family income is associated with a 2.98–fold increase in the likelihood of depressive symptoms (95% CI 1.86–4.77) compared to women belonging to the group with the highest family income. Regarding childhood adversities, women with a history of physical neglect in childhood were 1.59 times more likely to experience depressive symptoms (95% CI 1.16–2.16), and those who suffered sexual abuse were 1.57 times more likely (95% CI 1.03–2.39). Women with a history of childhood emotional abuse were 2.5 times more likely to experience depressive symptoms in adulthood (95% CI 1.82–3.44), as were participants who reported having a family member incarcerated, who were 1.90 times more likely to experience the outcome under study (95% CI 1.18–3.05).

**Table 3:** Raw and adjusted analysis of depressive symptomatology according to sociodemographic and economic data and types of childhood adversity among women residing in the Municipality of Vitória, ES, Brazil, 2022.

	OR	95% CI	p–Value	AOR	95% CI	p–Value
**Age range**			0.222			–
18 to 34 years old	*Ref.*			–	–	
35 to 59 years old	0.76	0.55–1.06		–	–	
≥60 years	0.76	0.52–1.12		–	–	
**Race–color**			0.412			–
White	*Ref.*			–	–	
Black	1.25	0.85–1.83		–	–	
Brown and others	0.97	0.71–1.33		–	–	
**Women’s education**			0.035			0.250
0 to 4 years	1.94	1.12–3.35		1.60	0.92–2.80	
5 to 8 years	1.30	0.87–1.94		0.99	0.65–1.51	
9 to 11 years old	*Ref.*			*Ref.*		
**Family income**			<0.001			<0.001
1st quintile (poorest)	2.98	1.86–4.77		2.98	1.86–4.77	
2nd quintile	2.25	1.39–3.65		2.25	1.39–3.65	
3rd quintile	1.30	0.78–2.15		1.30	0.78–2.15	
4th quintile	1.64	1.00–2.71		1.64	1.00–2.71	
5th quintile (richest)	*Ref.*			*Ref.*		
**Emotional Neglect**			<0.001			0.107
No	*Ref.*			*Ref.*		
Yes	1.75	1.29–2.38		1.31	0.94–1.82	
**Physical Neglect**			<0.001			0.003
No	*Ref.*			*Ref.*		
Yes	2.20	1.65–2.93		1.59	1.16–2.16	
**Sexual Abuse**			<0.001			0.037
No	*Ref.*			*Ref.*		
Yes	2.28	1.55–3.36		1.57	1.03–2.39	
**Physical Abuse**			<0.001			0.375
No	*Ref.*			*Ref.*		
Yes	2.02	1.52–2.70		1.17	0.83–1.64	
**Emotional Abuse**			<0.001			<0.001
No	*Ref.*			*Ref.*		
Yes	3.03	2.24–4.12		2.50	1.82–3.44	
**Parental Separation**			0.006			0.786
No	*Ref.*			*Ref.*		
Yes	1.48	1.12–1.96		1.04	0.77–1.41	
**Incarcerated relative**			<0.001			0.008
No	*Ref.*			*Ref.*		
Yes	2.99	1.91–4.61		1.90	1.18–3.05	
**Family member with mental health problems**			<0.001			0.106
No	*Ref.*			*Ref.*		
Yes	1.79	1.28–2.50		1.35	0.94–1.93	
**Alcohol abuse by family members**			<0.001			0.813
No	*Ref.*			*Ref.*		
Yes	1.89	1.41–2.53		0.96	0.67–1.37	

95% CI: 95% confidence interval.

p-value Pearson’s chi-squared test.

OR: Raw Odds Ratio (without adjustments).

ORa: Adjusted Odds Ratio.

## DISCUSSION

This study highlighted the importance of identifying vulnerability factors associated with women’s mental health, contributing to the planning of nursing care actions and the implementation of strategies for the early detection of depressive symptoms.

Note the loss of statistical significance of schooling after adjusting the variables in the model (p = 0.250). This change suggests an interrelationship with other documented factors, such as income. According to studies, education improves job market entry and economic self-sufficiency, which in turn reduces exposure to chronic stressors and increases women’s negotiating skills and autonomy in their social contexts, mitigating the likelihood of developing depressive symptoms and increasing real access to means of protection against stressors^(22,23)^.

In line with this, our findings showed that women in the lowest income quintile were 2.98 times more likely to exhibit depressive symptoms compared to those in the highest income bracket. This association suggests greater exposure to chronic psychosocial stressors among lower-income women—unfavorable living conditions, less social support, and limited access to education and medication—factors known to be associated with the development of depression^(24)^.

In the Brazilian context, marked by high social inequality and fragile safety nets, income is an important indicator of material deprivation, reflecting the barriers that overburden women’s coping capacity and increasing the likelihood of developing depressive symptoms. This finding is also found in international contexts, as presented in a cross-sectional study using data from the US National Health and Nutrition Examination Survey, which pointed to an association between income and depression^(25)^.

Regarding childhood adversity, physical neglect in childhood increased the odds of depressive symptoms by 1.59 times (95% CI 1.16–2.16). This finding is consistent with another study that identified psychological abuse and neglect associated with depression^(26)^. Unlike direct forms of violence, physical neglect deprives a child of basic care, generating feelings of loneliness and social isolation. This deficiency, which begins at this stage, compromises the development of social skills and emotional regulation, contributing to the emergence of depressive disorder in adulthood^(27)^.

Emotional abuse stood out as the strongest predictor among all the adversities investigated (ORa: 2.50; 95% CI 1.82–3.44). Although it is one of the most “silent” forms of child maltreatment, its strong association with depression in adulthood is related to vulnerability to developing a negative cognitive style, which leads to an increased risk of depression^(28,29)^. This is due to the often degrading, humiliating, and psychologically terrifying exposure to the caregiver, leading to feelings of helplessness and reduced self-esteem in the child. This culminates in a negative cognitive style that is also present in adulthood, and is a significant factor in the development of depression^(25)^. Furthermore, from the perspective of Attachment Theory, the lack of emotional support^(30)^ and the establishment of negative attachment predisposes individuals to the internalization of symptoms, contributing to the later development of depressive symptoms in life^(31)^.

Regarding sexual abuse (ORa 1.57; 95% CI 1.03–2.39), evidence suggests that sexual abuse can become “biologically embedded” in the organism through alterations in stress response pathways. Dysregulation of the hypothalamic-pituitary-adrenal (HPA) axis, and consequently increased cortisol reactivity, directly contribute to the development and maintenance of depressive symptoms^(32,33)^. It should be noted that this association is particularly strong in cases of atypical depression (hypersomnia and weight gain) and is linked to feelings of entrapment and habituation to pain, leading to risks of depressive behaviors^(34)^.

The incarceration of a family member nearly doubled the chance of depressive symptoms (95% CI 1.18–3.05). This finding reveals that the imprisonment of a family member is not an isolated event, but rather a trigger for a series of structural vulnerabilities, impacting the child’s social, economic, and health issues. Furthermore, parental absence can increase the likelihood of poor monitoring and supervision, raising the chances of risky behaviors and the possibility of substitute caregivers. This reconfiguration within the family unit enables other forms of mistreatment, including sexual abuse, thus creating a scenario of high emotional toxicity and a greater likelihood of developing depressive symptoms^(35)^.

These changes within the family unit, marked by ongoing conflicts and feelings of abandonment, act as potent chronic stressors that compromise the infant’s relational and social skills^(36)^. Furthermore, this stressful intrafamilial situation generates a breakdown of trust and the emergence of insecurity in the home environment, which can also hinder cognitive development, causing psychological damage and academic difficulties^(37)^.

The evidence presented here reinforces the urgency of strategies for identifying ACE, as they constitute a serious public health problem with persistent negative impacts throughout life. In this context, Nursing plays a strategic role due to its close relationship with communities and the support it provides to women throughout their life cycle.

Because nurses are often the professionals who establish the first contact with users of health services, they occupy a privileged position for the early identification of vulnerabilities. The quality of the patient-nurse relationship is a critical differentiator, as it creates an environment of trust, establishing a bond that encourages the expression of complaints, the sharing of stories, etc., allowing an understanding that depression is related to experiences that occurred in childhood. Thus, the professional can plan interventions, focus on promoting health to protect the child, and make timely referrals for psychosocial care. It is suggested that future studies investigate these specific interactions of adversity and whether material deprivation actually amplifies related trauma.

As a limitation of the study, we can point out the causality between the variables studied, as well as the selection bias, given that only women who had or had had an intimate partner in the last 24 months were included, which could limit the generalizability of the findings to the female population in general. Another aspect to highlight is that of memory, since self-reports were used when introducing questions about childhood to adults, and some information may have been forgotten by the participant. Furthermore, there is an information bias because, given the sensitive nature of the topic, the women interviewed might have difficulty accurately providing the information, which could lead to a possible underestimation of the available data. However, to minimize this possibility, the research was conducted privately, with only the woman and the researcher present, ensuring greater comfort for the interviewee.

## CONCLUSION

This study reaffirms the association between exposure to childhood adversities (emotional and sexual abuse, family confinement, and physical neglect) and socioeconomic deprivation as contexts of greater vulnerability to depressive symptoms in adult women. The findings are highly relevant as they provide robust population-based evidence in a Brazilian capital city, something that has been little explored in national studies.

The results highlight the importance of prevention and early intervention strategies and the role of nurses in this process, considering that they are often the professionals who establish the first contact with the user, holding a strategic position for early screening of vulnerabilities. Establishing an approach focused on protecting childhood, strengthening family ties, and reducing social inequalities, coupled with a comprehensive and sensitive perspective in health services, creates a paradigm for women’s and children’s health care, mitigating the deleterious effects of the situations experienced.

Finally, the findings reveal how important and necessary it is to invest in intersectoral public policies for the protection of childhood and the tackling of economic inequalities, to minimize adverse events in this important phase of life, since they negatively impact health throughout life.

## Data Availability

The entire dataset supporting the results of this study is available upon request from the corresponding author.
