# Supplementary material for: Childhood adversities and depressive symptoms in women: a population-based cross-sectional study
Source: Rev Esc Enferm USP. 2026 Sep 28;60:e20260138. doi: 10.1590/1980-220X-REEUSP-2026-0138en (PMC13638202; doi:10.1590/1980-220X-REEUSP-2026-0138en)
Supplement: Translation

# Translation: Adversidades na infância e sintomas depressivos em mulheres: estudo transversal de base populacional*

**DOI:** 10.1590/1980-220X-REEUSP-2026-0138pt

**Authors:** Tiffani Matos Oliveira, Franciele Marabotti Costa Leite

**Affiliations:** 1 Universidade Federal do Espírito Santo, Programa de Pós-Graduação em Saúde Coletiva, Vitória, ES, Brasil.

**Keywords:** Experiências adversas na infância, Depressão, Epidemiologia, Mulheres.

## Abstract

**Objetivo:**

Verificar a associação entre a exposição às adversidades na infância e a ocorrência de sintomas de depressão em mulheres na idade adulta.

**Método:**

Estudo epidemiológico, transversal, de base populacional com 1100 mulheres de Vitória, Espírito Santo, em 2022. Utilizou-se o Questionário Internacional de Adversidades na Infância e Inventario de Depressão de Beck. Nas análises, calcularam-se frequências brutas, e relativas, com intervalos de confiança de 95%. Nas análises bivariadas aplicou-se o teste qui-quadrado de Pearson, e, para as análises de associações com ajustes das variáveis de confusão, a Regressão logística. As análises foram realizadas no Stata 17.0.

**Resultados:**

Mulheres com histórico infantil de negligência física (ORa: 1,59; IC95%:1,16–2,16), abuso sexual (ORa: 1,57; IC95%:1,03–2,39) e algum familiar encarcerado (ORa: 1,90; IC95%:1,18–3,05) apresentaram maiores chances de sintomas depressivos durante a idade adulta quando comparadas àquelas que não vivenciaram tais experiencias.

**Conclusão:**

Os achados revelam o quão importante e necessário é o investimento em políticas públicas intersetoriais para proteção da infância e enfrentamento das desigualdades econômicas, de modo a minimizar eventos adversos nessa importante fase da vida, uma vez que impactam negativamente na saúde ao longo da vida.

## INTRODUÇÃO

As experiências adversas na infância (EAI) são um grande problema de saúde pública e englobam um conjunto de condições contextuais como perdas interpessoais, ambiente familiar disfuncional, maus-tratos (físicos, psicológicos e sexuais e negligências), dificuldades econômicas, violência comunitária^(1,2)^.

Dados internacionais indicam que milhões de crianças anualmente são expostas a EAI^(3)^, com prevalências significativas sendo encontradas em países da Europa e América do Norte^(4)^. Nos Estados Unidos, especificamente, dados de 2022 revelaram que mais de 3 milhões de crianças foram submetidas a maus-tratos, e ocorreram 1990 mortes registradas com relação direta com essas experiências^(5)^.

No contexto brasileiro, nas regiões Sul e Sudeste, as prevalências são igualmente alarmantes, com a ocorrência de pelo menos uma EAI variando de 53,6% a 85%^(6,7,8)^. Estudo realizado em Pelotas, no Rio Grande do Sul, identificou que 85% dos jovens de até 18 anos sofreram ao menos uma EAI, com maior incidência entre as meninas, que sofrem quatro ou mais tipos^(9)^. Diante desse cenário, a literatura salienta a importância de estudos no contexto brasileiro, considerando as particularidades socioeconômicas do país e a escassez de dados sobre essa temática^(10)^.

Considerando essas particularidades e a carência de dados populacionais sobre o tema, torna-se fundamental compreender como tais vivências são incorporadas pelo organismo feminino. É reconhecido que as experiências precoces de adversidades na infância promovem alterações estruturais e funcionais no desenvolvimento cerebral, as quais podem desregular os sistemas endócrinos e imunológicos. Essas modificações biológicas aumentam a suscetibilidade a uma série de condições clínicas e manifestações psicológicas, como alguns espectros da psicose, ansiedade e depressão^(9,11)^.

No caso específico da depressão, a exposição a maus-tratos na infância pode elevar de duas a três vezes mais o risco de surgimento do transtorno depressivo na vida adulta, sendo o abuso emocional e a negligência preditores fortes para esse desfecho^(12)^. Essa associação é mediada por uma interação complexa entre mecanismos biológicos (eixo hipotálamo-hipófise-adrenal (HPA), estrutura e função neural) e psicossociais (apego e habilidade de enfrentamento, estresse..); por um lado, ocorre a desregulação do eixo HPA e alterações na estrutura e função neural; por outro lado, consolidam-se padrões de apego inseguro e dificuldades nas habilidades de enfrentamento ao estresse. Ao moldarem a resposta emocional desde fases iniciais do desenvolvimento, esses fatores estabelecem uma vulnerabilidade que perdura ao longo de toda vida do indivíduo^(13,14)^.

A gravidade dessa associação torna-se ainda mais relevante devido ao cenário epidemiológico da patologia, que afeta quase 280 milhões de pessoas, e é um dos transtornos mais prevalentes e uma das principais causas de incapacidade no mundo^(15)^. Nesse cenário, a atuação do enfermeiro nos serviços de saúde é de grande relevância, visto que esse profissional frequentemente estabelece o primeiro contato com os usuários, favorecendo a identificação precoce de sintomas depressivos e o acompanhamento durante o processo de cuidado. A qualidade da relação paciente-enfermeiro pode contribuir para a expressão das queixas emocionais e a adesão às intervenções propostas^(16)^.

Diante do exposto, compreender a relação entre as adversidades na infância e a depressão torna-se imperativo para o direcionamento de estratégias de prevenção e cuidado no âmbito da saúde, uma vez que apesar do crescente número de estudos internacionais sobre a relação entre adversidades na infância e desfechos em saúde mental, ainda são limitadas as investigações de base populacional, especialmente voltadas à população feminina. Assim sendo, este estudo teve por objetivo verificar a associação entre a exposição às adversidades na infância e a ocorrência de sintomas de depressão em mulheres na idade adulta em Vitória, Espírito Santo.

## MÉTODO

### Desenho do Estudo

Trata-se de um estudo epidemiológico, observacional do tipo transversal, realizado a partir da pesquisa de campo intitulada “Violência contra a mulher em Vitória, Espírito Santo: Um estudo de base populacional”, realizada no ano de 2022.

### Local

O município de Vitória, Espírito Santo, possuía em 2021 aproximadamente uma população feminina estimada em 163.853, 49,98% da população de Vitória, de acordo com o Instituto Brasileiro de Geografia e Estatística (IBGE).

### População

A amostra foi composta por mulheres com idade de 18 anos ou mais residentes no município de Vitória, Espírito Santo, que tinham ou tiveram parceiros nos últimos 24 meses.

### Critérios de Seleção

Como critério para a inclusão na pesquisa foi estabelecido que as participantes deveriam ter idade igual ou superior a 18 anos, que tiveram ou tinham parceiro íntimo nos 24 meses anteriores à entrevista. Mulheres que não tinham capacidade de compreensão ou comunicação devido a déficit intelectual ou sensorial e que, portanto, não conseguiriam responder ao questionário da pesquisa^(17)^ foram excluídas.

### Definição da Amostra

A amostragem foi realizada em múltiplos estágios. Inicialmente, foi selecionada a unidade primária de amostragem, sendo esta os setores censitários do município de Vitória fornecidos pelo censo 2010 do IBGE. O total de domicílios na zona urbana de Vitória, Espírito Santo, é de 108.515, que foi dividido por 100 (número de setores a serem visitados) para se obter o pulo sistemático (1.085), respeitando-se probabilidade proporcional ao número de domicílios e mulheres dentro de cada setor. Após selecionar os setores censitários, foi feita uma lista por nível socioeconômico e sorteou-se de modo aleatório o número 513 (entre 1 e 1.085) pelo programa estatístico R, correspondendo ao número pertencente ao primeiro setor definido. Por fim, selecionaram-se os demais setores (99), o que ocorreu por meio da soma do pulo sistemático do setor inicial (184), até o término da listagem. A partir da seleção dos setores censitários, ocorreu a seleção dos domicílios (de forma aleatória) de acordo com a listagem disponível na plataforma on-line do IBGE. Após ser feita a listagem de mulheres elegíveis aos critérios do estudo, foram sorteadas as mulheres a responder a pesquisa. Para a realização do cálculo do tamanho da amostra, levou-se em consideração o nível de confiança de 95%, poder de 80% e razão exposto/não exposto de 1:1. A esse valor foram acrescidos 10% a título de perdas, e 30% para fatores de confusão, chegando a um tamanho amostral de 1.100 mulheres^(17)^.

### Coleta de Dados

A pesquisa realizou um estudo piloto em dezembro de 2021 e a coleta de dados ocorreu entre os meses de janeiro à maio de 2022, realizada por uma equipe de entrevistadoras devidamente treinadas, e contando com supervisores de campo. A coleta de dados foi realizada nos domicílios, por meio da utilização de tablets, respeitando-se a privacidade e o sigilo das mulheres^(17)^. O questionário foi estruturado por meio do REDCAP (Research Electronic Data Capture).

### Variáveis

A pesquisa utilizou como variáveis independentes as questões socioeconômicas e experiências familiares: Fatores socioeconômicos: Faixa etária (18 a 34 anos; 35 a 59 anos; ≥60 anos); Escolaridade da mulher (0 a 4 anos; 5 a 8 anos; 9 a 11 anos); Raça/cor (branca; preta; parda e outros); Renda familiar (1º quintil – mais pobres); 2º quintil; 3º quintil; 4º quintil e 5º quintil - mais ricas); Experiências familiares (Adversidades na infância): Abusos (físicos, sexuais e emocionais – não/sim); Negligência (físicas e emocionais – não/sim); Conflitos familiares (separação parental; familiar encarcerado; familiar com problemas de saúde mental; abuso de álcool por familiares - não/sim).

Para a avaliação das EAI foi utilizado o Questionário Internacional de Adversidades na Infância (ACE-IQ)^(2)^, validado no Brasil^(18)^. O instrumento tem por finalidade identificar e avaliar a exposição pregressa a 13 categorias diferentes de EAI, incluindo abuso sexual, emocional e físico, negligência emocional e física, violência familiar, uso de álcool/drogas, doença mental ou suicídio no âmbito domiciliar, envolvimento da família com atividades criminais, separação ou divórcio parental, dentre outras formas, de maneira a realizar uma avaliação da ocorrência e frequência de experiências adversas na infância^(18)^.

A variável de desfecho em estudo, sintomatologia depressiva, foi rastreada por meio da aplicação no instrumento do Inventário de Depressão de Beck (BDI) validado no Brasil^(19)^. A BDI é uma medida de autorrelato composta por 21 itens, em que cada item contém quatro afirmativas. Estas têm graus crescentes de intensidade de sintomas depressivos, com escores variando de 0 a 3 em escala tipo Likert^(20)^.

A escala possui questões sobre: Tristeza; Pessimismo; Sentimento de fracasso; Insatisfação; Culpa; Punição; Autoaversão; Autoacusações; Ideias suicidas; Choro; Irritabilidade; Retraimento social; Indecisão; Mudança na autoimagem; Dificuldade para trabalhar; Insônia; Fatigabilidade; Perda de apetite; Perda de peso; Preocupações somáticas; e Perda de libido. Assim, a escala é utilizada como uma forma de rastrear sintomas depressivos em populações com ou sem diagnóstico de depressão, objetivando a identificação da intensidade dos sintomas depressivos^(20)^.

### Análise e Tratamento dos Dados

A análise estatística iniciou-se pela avaliação exploratória dos escores do BDI. A verificação de normalidade, realizada por meio de histograma e confirmada pelo teste de Shapiro-Wilk (*p* < 0,001), indicou assimetria e não normalidade dos dados. Diante disso, optou-se pela categorização da variável desfecho com base nos pontos de corte de Cunha: 0 a 11 para sintomatologia mínima; 12 a 19 para leve; 20 a 35 para moderada; e 36 a 63 para sintomas graves, procedendo-se à dicotomização da variável, definindo-se como presença de sintomatologia depressiva os escores iguais ou superiores a 12, enquanto pontuações de 0 a 11 foram classificadas como sem sintomatologia depressiva^(19)^.

Os dados foram analisados por meio de estatística descritiva por frequência bruta, relativa e seus intervalos de confiança de 95% (IC95%). Para as análises bivariadas foram realizados teste qui-quadrado de Pearson (χ^2^), considerando significância estatística de 5%. A associação entre as variáveis foi feita por meio da Regressão Logística e os seus respectivos intervalos de confiança (IC95%). Foram considerados, para entrar na análise ajustada, valores de p < 0,20 da bivariada e a permanência no ajuste quando p < 0,05.

Adotou-se um modelo hierárquico analítico de entrada das variáveis, fundamentado no modelo teórico de determinação social da saúde^(21)^. O modelo analítico ocorreu em dois níveis, sendo o primeiro (distal) as adversidades na infância (negligências, abusos, familiar encarcerado, etc.), seguido do nível intermediário os dados sociodemográficos (escolaridade, renda), os quais impactam diretamente no desfecho (Figura 1). As análises foram realizadas no programa estatístico Stata versão 17.0.

**Figura 1:** Amostra de análise.

Fonte: elaborada pelas autoras, 2025.

### Aspectos Ético

O projeto foi submetido ao Comitê de Ética em Pesquisa da Universidade Federal do Espírito Santo, e aprovado por meio do parecer n° 4.974.080, de forma a atender a resolução n°466/2012. Todas as mulheres entrevistadas assinaram o Termo de Compromisso Livre e Esclarecido (TCLE).

## RESULTADOS

Observou-se uma relação estatisticamente significativa entre as condições socioeconômicas e a sintomatologia depressiva (p < 0,05). Mulheres pertencentes ao grupo de menor renda familiar apresentaram maior frequência de sintomatologia depressiva (34,5%), assim como aquelas participantes com menores anos de escolaridade (36,1%) (Tabela 1).

**Tabela 1:** Análise bivariada entre a sintomatologia depressiva com dados sociodemográficos e econômicos entre as mulheres residentes no município de Vitória, ES, Brasil, 2022.

	N (%)	IC95%	p valor
**Faixa etária**			0,221
18 a 34 anos	82 (27,6)	22,8–33,0	
35 a 59 anos	119 (22,6)	19,2–26,4	
≥60 anos	59 (22,5)	17,9–27,8	
**Raça–cor**			0,419
Branca	102 (23,4)	19,5–27,6	
Negra	54 (27,6)	21,7–34,2	
Parda e outros	104 (22,9)	19,3–27,0	
**Escolaridade da mulher**			0,033
0 a 4 anos	22 (36,1)	25,1–48,8	
5 a 8 anos	39 (27,5)	20,7–35,4	
9 a 11 anos	199 (22,5)	19,9–25,4	
**Renda familiar**			<0,001
1º quintil (20% com menor renda)	77 (34,5)	28,6–41,0	
2º quintil	61 (28,5)	22,9–34,9	
3º quintil	43 (18,7)	14,2–24,3	
4º quintil	48 (22,5)	17,4–28,6	
5º quintil (20% com maior renda)	175 (15,1)	10,8–20,6	

N: frequência bruta e %: frequência relativa.

IC95%: Intervalo de confiança de 95%.

p-valor teste qui-quadrado de Pearson.

Os resultados da análise bivariada (Tabela 2) revelaram que a exposição a todas as categorias das adversidades na infância estiveram relacionadas à ocorrência de sintomas depressivos na idade adulta (p < 0,05).

**Tabela 2:** Distribuição dos sintomas depressivos entre mulheres residentes no Município de Vitória, ES, de acordo com os tipos de adversidades na infância – Vitória, ES, Brasil, 2022.

	N	%	IC95%	p-valor*
**Negligência Emocional**				<0,001
Não	174	21,3	18,6–24,2	
Sim	86	32,1		
**Negligência Física**				<0,001
Não	139	19,0	16,3–22,0	
Sim	121	34,1	29,3–39,2	
**Abuso Sexual**				<0,001
Não	210	21,9	19,4–24,6	
Sim	50	39,1	31,0–47,8	
**Abuso Físico**				<0,001
Não	94	17,6	14,6–21,0	
Sim	166	30,1	26,4–34,1	
Abuso Emocional				<0,001
Não	71	13,9	11,2–17,2	
Sim	189	32,9	29,1–36,8	
**Separação Parental**				0,006
Não	118	20,6	17,4–24,1	
Sim	142	27,7	24,0–31,8	
**Familiar encarcerado**				<0,001
Não	219	22,0	19,5–24,7	
Sim	41	45,6	35,6–55,8	
**Familiar com problemas de saúde mental**				0,001
Não	193	21,8	19,2–24,7	
Sim	67	33,3	27,2–40,1	
**Abuso de álcool por familiares**				<0,001
Não	155	20,3	17,6–23,3	
Sim	105	32,5	27,6–37,8	

N: frequência bruta.

%: frequência relativa.

IC95%: Intervalo de confiança de 95%.

p-valor teste qui-quadrado de Pearson.

Após a análise ajustada (Tabela 3), nota-se que pertencer ao primeiro quintil de renda familiar está associado a um aumento de 2,98 vezes mais chances de sintomatologia depressiva (IC95% 1,86–4,77) em comparação às mulheres que pertencem ao grupo com renda familiar mais alta. No que tange às adversidades na infância, mulheres com histórico de negligência física na infância apresentaram um aumento de 1,59 vezes mais chances de sintomatologia depressiva (IC95% 1,16–2,16) e aquelas que sofreram abuso sexual apresentaram 1,57 vezes mais chances (IC95%1,03–2,39). Mulheres com histórico na infância de abuso emocional apresentaram 2,5 vezes mais chance de apresentarem sintomatologia depressiva na idade adulta (IC95% 1,82–3,44), assim como as participantes que referiram ter algum familiar encarcerado apresentaram chances 1,90 vezes maiores do desfecho em estudo (IC95% 1,18–3,05).

**Tabela 3:** Análise bruta e ajustada da sintomatologia depressiva de acordo com os dados sociodemográficos, econômicos e os tipos de adversidades na infância entre as mulheres residentes no Município de Vitória, ES, Brasil, 2022.

	OR	IC95%	p–valor	AOR	IC95%	p–valor
**Faixa etária**			0,222			–
18 a 34 anos	*Ref.*			–	–	
35 a 59 anos	0,76	0,55–1,06		–	–	
≥60 anos	0,76	0,52–1,12		–	–	
**Raça–cor**			0,412			–
Branca	*Ref.*			–	–	
Negra	1,25	0,85–1,83		–	–	
Parda e outros	0,97	0,71–1,33		–	–	
**Escolaridade da mulher**			0,035			0,250
0 a 4 anos	1,94	1,12–3,35		1,60	0,92–2,80	
5 a 8 anos	1,30	0,87–1,94		0,99	0,65–1,51	
9 a 11 anos	*Ref.*			*Ref.*		
**Renda familiar**			<0,001			<0,001
1º quintil (mais pobres)	2,98	1,86–4,77		2,98	1,86–4,77	
2º quintil	2,25	1,39–3,65		2,25	1,39–3,65	
3º quintil	1,30	0,78–2,15		1,30	0,78–2,15	
4º quintil	1,64	1,00–2,71		1,64	1,00–2,71	
5º quintil (mais ricos)	*Ref.*			*Ref.*		
**Negligência Emocional**			<0,001			0,107
Não	*Ref.*			*Ref.*		
Sim	1,75	1,29–2,38		1,31	0,94–1,82	
**Negligência Física**			<0,001			0,003
Não	*Ref.*			*Ref.*		
Sim	2,20	1,65–2,93		1,59	1,16–2,16	
**Abuso Sexual**			<0,001			0,037
Não	*Ref.*			*Ref.*		
Sim	2,28	1,55–3,36		1,57	1,03–2,39	
**Abuso Físico**			<0,001			0,375
Não	*Ref.*			*Ref.*		
Sim	2,02	1,52–2,70		1,17	0,83–1,64	
**Abuso Emocional**			<0,001			<0,001
Não	*Ref.*			*Ref.*		
Sim	3,03	2,24–4,12		2,50	1,82–3,44	
**Separação Parental**			0,006			0,786
Não	*Ref.*			*Ref.*		
Sim	1,48	1,12–1,96		1,04	0,77–1,41	
**Familiar encarcerado**			<0,001			0,008
Não	*Ref.*			*Ref.*		
Sim	2,99	1,91–4,61		1,90	1,18–3,05	
**Familiar com problemas de saúde mental**			<0,001			0,106
Não	*Ref.*			*Ref.*		
Sim	1,79	1,28–2,50		1,35	0,94–1,93	
**Abuso de álcool por familiares**			<0,001			0,813
Não	*Ref.*			*Ref.*		
Sim	1,89	1,41–2,53		0,96	0,67–1,37	

IC95%: Intervalo de confiança de 95%.

p-valor teste qui-quadrado de Pearson.

OR: Odds Ratio bruto (sem ajustes).

ORa: Odds Ratio ajustado.

## DISCUSSÃO

O presente estudo evidenciou a importância da identificação de fatores de vulnerabilidade associados à saúde mental das mulheres, contribuindo para o planejamento de ações de cuidado em enfermagem e para a implementação de estratégias de detecção precoce de sintomas depressivos.

Nota-se a perda de significância estatística da escolaridade após o ajuste das variáveis no modelo (p = 0,250). Essa mudança sugere uma inter-relação com outros fatores documentados, como a renda. Segundo estudos, a educação qualifica a inserção no mercado de trabalho e a autossuficiência econômica, as quais por sua vez reduzem a exposição a estressores crônicos e aumentam a capacidade de negociação e autonomia da mulher em seus contextos sociais, mitigando a probabilidade do surgimento de sintomas depressivos e aumentando o acesso real a meios de proteção contra estressores^(22,23)^.

Em consonância com isso, nossos achados observaram que as mulheres pertencentes ao quintil de menor renda apresentaram chances 2,98 vezes maiores de manifestarem sintomatologia depressiva em comparação às de maior renda. Tal associação sugere maior exposição a estressores psicossociais crônicos entre as mulheres de menor renda - condições de vida desfavoráveis, menor apoio social e acesso limitado à educação e medicamentos - fatores reconhecidamente associados ao desenvolvimento da depressão^(24)^.

No contexto brasileiro, marcado por alta desigualdade social e redes de proteção frágeis, a renda configura-se como um importante indicador de privação material, refletindo nas barreiras que sobrecarregam a capacidade de enfrentamento das mulheres, elevando a possibilidade ao desfecho da sintomatologia depressiva. Esse achado também é encontrado em contextos internacionais, como apresentado em um estudo transversal utilizando dados do Inquérito Nacional de Saúde e Nutrição dos EUA, que apontou uma associação entre renda e depressão^(25)^.

Concernente às adversidades na infância, a negligência física na infância aumentou em 1,59 vezes as chances de sintomatologia depressiva (IC95% 1,16–2,16). Esse achado vai ao encontro de outro estudo que identificou o abuso psicológico e a negligência associados à depressão^(26)^. Diferente de formas diretas de violência, a negligência física priva a criança de cuidados básicos, gerando sentimentos de solidão e isolamento social. Essa carência que começa nessa fase compromete o desenvolvimento de habilidades sociais e da regulação emocional, que contribui para o surgimento do transtorno depressivo na fase adulta^(27)^.

O abuso emocional destacou-se como preditor de maior magnitude entre todas as adversidades investigadas (ORa: 2,50; IC95% 1,82–3,44). Embora seja uma das formas mais “silenciosas” de maus-tratos infantis, sua forte associação com a depressão na vida adulta está relacionada à vulnerabilidade ao desenvolvimento de um estilo cognitivo negativo, o que acarreta um aumento do risco de depressão^(28,29)^. Isso se deve à exposição muitas vezes degradante, humilhante e aterrorizante psicologicamente ao cuidador, levando a sentimentos de impotência e autoestima reduzidos da criança. Isso culmina em um estilo cognitivo negativo apresentado também na fase adulta, sendo um fator importante para o surgimento da depressão^(25)^. Ademais, sob a ótica da Teoria do Apego, a falta de apoio emocional^(30)^ e o estabelecimento de apego negativo predispõem à internalização de sintomas, contribuindo para posteriormente apresentarem quadros depressivos na vida^(31)^.

Quanto ao abuso sexual (ORa 1,57; IC95% 1,03–2,39), evidências sugerem que o abuso sexual pode tornar-se “biologicamente incorporado” ao organismo por meio de alterações nas vias de resposta ao estresse. A desregulação do eixo hipotálamo hipófise-adrenal (HPA), e consequentemente maior reatividade do cortisol, contribuem diretamente para o desenvolvimento e a manutenção dos sintomas depressivos^(32,33)^. Destaca-se que essa associação é particularmente robusta em casos de depressão atípica (hipersonia e ganho de peso) e está vinculada a sentimentos de aprisionamento e habituação à dor, levando a riscos de comportamentos depressivos^(34)^.

O encarceramento de um familiar aumentou em quase duas vezes a chance de sintomatologia depressiva (IC95% 1,18–3,05). Esse achado revela que a prisão de um membro da família não é um evento isolado, mas desencadeador de uma série de vulnerabilidades estruturais, impactando em questões sociais, econômicas e de saúde da criança. Além disso, a ausência parental pode aumentar as chances de baixo monitoramento e da supervisão parental, elevando as chances de comportamentos de risco, e a possibilidade da presença de substitutos. Essa reconfiguração no núcleo familiar possibilita outras formas de maus-tratos, incluindo abuso sexual, e dessa forma proporcionando um cenário de alta toxicidade emocional e maiores chances do surgimento de sintomas depressivos^(35)^.

Tais alterações no núcleo familiar, marcadas por conflitos contínuos e sentimentos de abandono, atuam como potentes estressores crônicos que comprometem as habilidades relacionais e sociais do infante^(36)^. Além disso, essa situação estressora intrafamiliar gera uma quebra de confiança e surgimento de insegurança no ambiente doméstico, podendo também atrapalhar o desenvolvimento cognitivo, acarretando danos psicológicos e prejuízos escolares^(37)^.

As evidências aqui apresentadas reforçam a urgência de estratégias para a identificação das EAI, uma vez que se constituem como um grave problema de saúde pública, com impactos negativos persistentes ao longo da vida. Nesse contexto, a Enfermagem desempenha um papel estratégico devido à sua atuação próxima às comunidades e o acompanhamento das mulheres ao longo do ciclo de vida.

O enfermeiro, por ser frequentemente o profissional que estabelece o primeiro contato com os usuários de serviços de saúde, ocupa uma posição privilegiada para a identificação precoce de vulnerabilidades. A qualidade da relação paciente enfermeiro é um diferencial crítico, pois cria um ambiente de confiança, estabelecendo um vínculo que favorece as expressões de queixas, revelações de histórias e etc, possibilitando uma compreensão de que a depressão tem relação com as experiências ocorridas na infância. Assim, o profissional pode planejar intervenções, focar na promoção de saúde para a proteção da criança e no encaminhamento oportuno para o cuidado psicossocial. Sugere-se que estudos futuros investiguem essas interações específicas de adversidades e se a privação material realmente amplifica os traumas relacionados.

Como limitação do estudo, podemos apontar a causalidade entre as variáveis estudadas, assim como o viés de seleção, uma vez que houve apenas a inclusão de mulheres que possuíam ou possuíram parceiro íntimo nos últimos 24 meses, o que poderia limitar a generalização dos achados para a população feminina em geral. Outro viés a destacar é o de memória, uma vez que foram utilizados autorrelatos ao se introduzir perguntas sobre a infância em adultos e alguma informação poderia ter sido esquecida pela participante. Além disso, há um viés de informação já que, por ser uma temática sensível, as mulheres entrevistadas poderiam ter dificuldade em precisar as informações fornecidas, o que poderia favorecer a uma possível subestimação das informações presentes. No entanto, para minimizar essa possibilidade, a pesquisa foi realizada em âmbito privado, com apenas a mulher e o pesquisador, garantindo maior conforto para a entrevistada.

## CONCLUSÃO

Este estudo reafirma a associação entre a exposição às adversidades na infância (abuso emocional e sexual, o encarceramento familiar e a negligência física) e a privação socioeconômica como contextos de maior vulnerabilidade à sintomatologia depressiva em mulheres adultas. Os achados são de grande relevância ao fornecer evidências robustas de base populacional em uma capital brasileira, algo ainda pouco explorado em estudos nacionais.

Os resultados apontam a importância de estratégias de prevenção e intervenção precoce e da atuação do enfermeiro nesse processo, considerando que frequentemente é o profissional que estabelece o primeiro contato com a usuária, tendo uma posição estratégica de rastreio precoce das vulnerabilidades. Ao ser estabelecido um enfoque voltado à proteção da infância, fortalecimento de vínculos familiares e à redução das desigualdades sociais, aliado a um olhar integral e sensível nos serviços de saúde, cria-se um paradigma de cuidado à saúde da mulher e da criança, mitigando-se os efeitos deletérios das situações vivenciadas.

Por fim, os achados revelam o quão importante e necessário é o investimento em políticas públicas intersetoriais para proteção da infância e enfrentamento das desigualdades econômicas, de modo a minimizar eventos adversos nessa importante fase da vida, uma vez que impactam negativamente na saúde ao longo da vida.

## Data Availability

Todo o conjunto de dados que dá suporte aos resultados deste estudo está disponível mediante solicitação ao autor correspondente.

# Translation: Adversidades en la infancia y síntomas depresivos en mujeres: un estudio transversal de base poblacional*

**DOI:** 10.1590/1980-220X-REEUSP-2026-0138es

**Authors:** Tiffani Matos Oliveira, Franciele Marabotti Costa Leite

**Affiliations:** 1 Universidade Federal do Espírito Santo, Programa de Pós-Graduação em Saúde Coletiva, Vitória, ES, Brasil.

**Keywords:** Experiencias Adversas de la Infancia, Depresión, Epidemiología, Mujeres

## Abstract

**Objetivo:**

Investigar la relación entre la exposición a la adversidad en la infancia y la aparición de síntomas depresivos en mujeres en la edad adulta.

**Método:**

Estudio epidemiológico, transversal, de base poblacional con 1100 mujeres de Vitória, Espírito Santo, en 2022. Se utilizaron el Cuestionario Internacional de Adversidades en la Infancia y el Inventario de Depresión de Beck. En los análisis se calcularon frecuencias brutas y relativas, con intervalos de confianza del 95%. En los análisis bivariados se aplicó la prueba de chi-cuadrado de Pearson, y para los análisis de asociaciones con ajustes por variables de confusión se utilizó la regresión logística. Los análisis se realizaron utilizando Stata 17.0.

**Resultados:**

Las mujeres con antecedentes de negligencia física en la infancia (ORa: 1,59; IC del 95%: 1,16–2,16), abuso sexual (ORa: 1,57; IC del 95%: 1,03–2,39) y un familiar encarcelado (ORa: 1,90; IC del 95%: 1,18–3,05) tenían más probabilidades de experimentar síntomas depresivos en la edad adulta en comparación con aquellas que no habían tenido tales experiencias.

**Conclusión:**

Los resultados revelan la importancia y la necesidad de invertir en políticas públicas intersectoriales para la protección de la infancia y la lucha contra las desigualdades económicas, con el fin de minimizar los acontecimientos adversos en esta importante etapa de la vida, ya que repercuten negativamente en la salud a lo largo de toda la vida.

## INTRODUCCIÓN

Las experiencias adversas en la infancia (EAI) constituyen un gran problema de salud pública y abarcan un conjunto de condiciones contextuales como pérdidas interpersonales, un entorno familiar disfuncional, maltrato (físico, psicológico y sexual, así como negligencia), dificultades económicas y violencia comunitaria^(1,2)^.

Los datos internacionales indican que millones de niños y niñas están expuestos anualmente a EAI^(3)^, con prevalencias significativas en países de Europa y América del Norte^(4)^. En Estados Unidos, específicamente, datos de 2022 revelaron que más de 3 millones de niños y niñas sufrieron maltrato, y se registraron 1990 muertes directamente relacionadas con estas experiencias^(5)^.

En el contexto brasileño, en las regiones del Sur y del Sudeste, las prevalencias son igualmente alarmantes, con una incidencia de al menos un caso de maltrato infantil que varía entre el 53,6 % y el 85 %^(6,7,8)^. Un estudio realizado en Pelotas, en Río Grande del Sur, identificó que el 85 % de los jóvenes de hasta 18 años sufrieron al menos un caso de EAI, con mayor incidencia entre las niñas, quienes sufren cuatro o más tipos^(9)^. Ante este panorama, la literatura destaca la importancia de los estudios en el contexto brasileño, considerando las particularidades socioeconómicas del país y la escasez de datos sobre este tema^(10)^.

Teniendo en cuenta estas particularidades y la falta de datos poblacionales sobre el tema, resulta fundamental comprender cómo el organismo femenino asimila esas experiencias. Se reconoce que las experiencias tempranas de adversidades en la infancia provocan alteraciones estructurales y funcionales en el desarrollo cerebral, las cuales pueden desregular los sistemas endocrino e inmunológico. Estas modificaciones biológicas aumentan la susceptibilidad a una serie de afecciones clínicas y manifestaciones psicológicas, como ciertos espectros de psicosis, ansiedad y depresión^(9,11)^.

En el caso específico de la depresión, la exposición al maltrato durante la infancia puede aumentar de dos a tres veces el riesgo de desarrollar un trastorno depresivo en la edad adulta, siendo el abuso emocional y la negligencia fuertes predictores de este resultado^(12)^. Esta asociación está mediada por una interacción compleja entre mecanismos biológicos (eje hipotálamo-hipófisis-suprarrenal [HPA], estructura y función neural) y psicosociales (apego y capacidad de afrontamiento, estrés...); por un lado, se produce una desregulación del eje HPA y alteraciones en la estructura y función neural; por otro lado, se consolidan patrones de apego inseguro y dificultades en las habilidades para afrontar el estrés. Al moldear la respuesta emocional desde las primeras etapas del desarrollo, estos factores establecen una vulnerabilidad que perdura a lo largo de toda la vida del individuo^(13,14)^.

La gravedad de esta asociación cobra aún mayor relevancia debido al panorama epidemiológico de la patología, que afecta a casi 280 millones de personas y es uno de los trastornos más prevalentes y una de las principales causas de discapacidad en el mundo^(15)^. En este contexto, la labor del enfermero en los servicios de salud reviste gran importancia, ya que este profesional suele establecer el primer contacto con los usuarios, lo que favorece la identificación temprana de los síntomas depresivos y el acompañamiento durante el proceso de atención. La calidad de la relación paciente-enfermero puede contribuir a que el paciente exprese sus dificultades emocionales y a que se adhiera a las intervenciones propuestas^(16)^.

Ante lo expuesto, comprender la relación entre las adversidades en la infancia y la depresión se vuelve imprescindible para orientar las estrategias de prevención y atención en el ámbito de la salud, ya que, a pesar del creciente número de estudios internacionales sobre la relación entre las adversidades en la infancia y los resultados en salud mental, aún son limitadas las investigaciones de base poblacional, especialmente aquellas enfocadas en la población femenina.

Por lo tanto, el objetivo de este estudio fue verificar la asociación entre la exposición a adversidades en la infancia y la presencia de síntomas de depresión en mujeres adultas en Vitória, Espírito Santo.

## MÉTODO

### Diseño del Estudio

Se trata de un estudio epidemiológico, observacional y transversal, basado en la investigación de campo titulada “Violencia contra la mujer en Vitória, Espírito Santo: un estudio poblacional”, realizada en el año 2022.

### Lugar

El municipio de Vitória, Espírito Santo, contaba en 2021 con una población femenina estimada en 163 853, lo que representa el 49,98 % de la población de Vitória, según el Instituto Brasileño de Geografía y Estadística (IBGE).

### Población

La muestra estuvo compuesta por mujeres de 18 años o más residentes en el municipio de Vitória, Espírito Santo, que tenían o habían tenido parejas en los últimos 24 meses.

### Criterios de Selección

Como criterio para la inclusión en la investigación se estableció que las participantes debían tener 18 años o más y haber tenido o tener una pareja íntima en los 24 meses previos a la entrevista. Se excluyó a las mujeres que no tenían capacidad de comprensión o comunicación debido a un déficit intelectual o sensorial y que, por lo tanto, no podrían responder al cuestionario de la investigación^(17)^.

### Definición de la Muestra

El muestreo se realizó en múltiples etapas. Inicialmente, se seleccionó la unidad primaria de muestreo, que fueron los sectores censales del municipio de Vitória proporcionados por el censo de 2010 del IBGE. El total de hogares en la zona urbana de Vitória, Espírito Santo, es de 108 515, cifra que se dividió entre 100 (número de sectores a visitar) para obtener el salto sistemático (1 085), respetando la probabilidad proporcional al número de hogares y de mujeres dentro de cada sector. Tras seleccionar los sectores censales, se elaboró una lista por nivel socioeconómico y se sorteó al azar el número 513 (entre 1 y 1 085) mediante el programa estadístico R, el cual correspondía al número perteneciente al primer sector definido. Por último, se seleccionaron los sectores restantes (99), lo cual se realizó sumando el salto sistemático del sector inicial (184) hasta el final de la lista. A partir de la selección de los sectores censales, se procedió a la selección de los hogares (de manera aleatoria) de acuerdo con la lista disponible en la plataforma en línea del IBGE. Una vez elaborada la lista de mujeres que cumplían con los criterios del estudio, se seleccionaron al azar a las mujeres que responderían la encuesta. Para calcular el tamaño de la muestra, se tomó en cuenta un nivel de confianza del 95 %, un poder estadístico del 80 % y una relación entre expuestas y no expuestas de 1:1.

A este valor se le sumó un 10 % para cubrir las pérdidas y un 30 % para los factores de confusión, lo que dio como resultado un tamaño de muestra de 1 100 mujeres^(17)^.

### Recolección de Datos

La investigación llevó a cabo un estudio piloto en diciembre de 2021 y la recolección de datos se realizó entre los meses de enero y mayo de 2022, a cargo de un equipo de entrevistadoras debidamente capacitadas y con el apoyo de supervisores de campo. La recolección de datos se llevó a cabo en los hogares, mediante el uso de tabletas, respetando la privacidad y la confidencialidad de las mujeres^(17)^. El cuestionario se estructuró mediante REDCAP (Research Electronic Data Capture).

### Variables

La investigación utilizó como variables independientes las cuestiones socioeconómicas y las experiencias familiares: Factores socioeconómicos: Grupo de edad (de 18 a 34 años; de 35 a 59 años; ≥60 años); Nivel educativo de la mujer (de 0 a 4 años; de 5 a 8 años; de 9 a 11 años); Raza/color (blanca; negra; mestiza y otras); Ingresos familiares (1.er quintil —los más pobres—; 2.º quintil; 3.er quintil; 4.º quintil y 5.º quintil — los más ricos—); Experiencias familiares (adversidades en la infancia): Abusos (físicos, sexuales y emocionales —no/sí—); Negligencia (física y emocional —no/sí—); Conflictos familiares (separación de los padres; familiar encarcelado; familiar con problemas de salud mental; abuso de alcohol por parte de familiares —no/sí—).

Para la evaluación de los EAI se utilizó el Cuestionario Internacional de Adversidades en la Infancia (ACE-IQ)^(2)^, validado en Brasil^(18)^. El instrumento tiene como objetivo identificar y evaluar la exposición previa a 13 categorías diferentes de EAI, incluyendo abuso sexual, emocional y físico; negligencia emocional y física; violencia familiar; consumo de alcohol o drogas; enfermedad mental o suicidio en el ámbito familiar; participación de la familia en actividades delictivas; separación o divorcio de los padres; entre otras formas, con el fin de evaluar la presencia y la frecuencia de experiencias adversas en la infancia^(18)^.

La variable de resultado analizada, la sintomatología depresiva, se evaluó mediante la aplicación del Inventario de Depresión de Beck (Beck Depression Inventory - BDI), validado en Brasil^(19)^. El BDI es una medida de autoinforme compuesta por 21 ítems, en la que cada ítem contiene cuatro afirmaciones. Estas presentan grados crecientes de intensidad de los síntomas depresivos, con puntuaciones que varían de 0 a 3 en una escala tipo Likert^(20)^.

La escala incluye preguntas sobre: Tristeza; pesimismo; sensación de fracaso; insatisfacción; culpa; castigo; auto aversión; autoacusaciones; ideas suicidas; llanto; irritabilidad; retraimiento social; indecisión; cambio en la autoimagen; dificultad para trabajar; insomnio; fatiga; pérdida de apetito; pérdida de peso; preocupaciones somáticas; y pérdida de la libido.

Así, la escala se utiliza como una forma de detectar síntomas depresivos en poblaciones con o sin diagnóstico de depresión, con el objetivo de identificar la intensidad de los síntomas depresivos^(20)^.

### Análisis y Procesamiento de los Datos

El análisis estadístico comenzó con una evaluación exploratoria de los puntajes del BDI. La verificación de la normalidad, realizada mediante un histograma y confirmada por la prueba de Shapiro-Wilk (*p* < 0,001), indicó asimetría y datos no normales. Ante esto, se optó por categorizar la variable de resultado basándose en los puntos de corte de Cunha: de 0 a 11 para sintomatología mínima; de 12 a 19 para síntomas leves; de 20 a 35 para síntomas moderados; y de 36 a 63 para síntomas graves, procediéndose a la dicotomización de la variable, definiendo como presencia de sintomatología depresiva las puntuaciones iguales o superiores a 12, mientras que las puntuaciones de 0 a 11 se clasificaron como sin sintomatología depresiva^(19)^.

Los datos se analizaron mediante estadística descriptiva, con frecuencias brutas y relativas, y sus intervalos de confianza del 95 % (IC95 %). Para los análisis bivariados se realizó la prueba de Chi-cuadrado de Pearson (χ^2^), considerando un nivel de significación estadística del 5 %. La asociación entre las variables se determinó mediante regresión logística y sus respectivos intervalos de confianza (IC95%). Para incluirlas en el análisis ajustado, se consideraron valores de p < 0,20 en el análisis bivariado y se mantuvieron en el ajuste cuando p < 0,05.

Se adoptó un modelo jerárquico analítico de entrada de variables, basado en el modelo teórico de determinación social de la salud^(21)^. El modelo analítico se estructuró en dos niveles: el primero (distal) abarcó las adversidades en la infancia (negligencia, abusos, familiar encarcelado, etc.), seguido del nivel intermedio, que incluyó los datos sociodemográficos (escolaridad, ingresos), los cuales impactan directamente en el desenlace (Figura 1). Los análisis se llevaron a cabo mediante el programa estadístico Stata versión 17.0.

**Figura 1:** Muestra de análisis.

Fuente: elaborada por las autoras, 2025.

### Aspectos Éticos

El proyecto se presentó ante el Comité de Ética en Investigación de la Universidad Federal de Espírito Santo y fue aprobado mediante el dictamen n.º 4.974.080, de conformidad con la resolución n.º 466/2012. Todas las mujeres entrevistadas firmaron el Término de Compromiso Libre e Informado (TCLE).

## RESULTADOS

Se observó una relación estadísticamente significativa entre las condiciones socioeconómicas y la sintomatología depresiva (p < 0,05). Las mujeres que pertenecían al grupo de menor ingreso familiar presentaron una mayor frecuencia de sintomatología depresiva (34,5 %), al igual que aquellas participantes con menos años de escolaridad (36,1 %) (Tabla 1).

**Tabla 1:** Análisis bivariado de la relación entre los síntomas depresivos y los datos sociodemográficos y económicos de las mujeres residentes en el municipio de Vitória, ES, Brasil, 2022.

	N (%)	IC del 95 %	Valor p
**Grupo de edad**			0,221
De 18 a 34 años	82 (27,6)	22,8–33,0	
De 35 a 59 años	119 (22,6)	19,2–26,4	
≥60 años	59 (22,5)	17,9–27,8	
**Raza y color de piel**			0,419
Blanca	102 (23,4)	19,5–27,6	
Negra	54 (27,6)	21,7–34,2	
Mestizas y otras	104 (22,9)	19,3–27,0	
**Nivel educativo de la mujer**			0,033
De 0 a 4 años	22 (36,1)	25,1–48,8	
De 5 a 8 años	39 (27,5)	20,7–35,4	
De 9 a 11 años	199 (22,5)	19,9–25,4	
**Renta familiar**			<0,001
1.º quintil (el 20 % con menores ingresos)	77 (34,5)	28,6–41,0	
2.º quintil	61 (28,5)	22,9–34,9	
3.º quintil	43 (18,7)	14,2–24,3	
4.º quintil	48 (22,5)	17,4–28,6	
5.º quintil (el 20 % con mayores ingresos)	175 (15,1)	10,8–20,6	

N: frecuencia bruta y %: frecuencia relativa.

IC95 %: Intervalo de confianza del 95 %.

Valor p de la prueba de chi-cuadrado de Pearson.

Los resultados del análisis bivariado (Tabla 2) revelaron que la exposición a todas las categorías de adversidades en la infancia se relacionaba con la aparición de síntomas depresivos en la edad adulta (p < 0,05).

**Tabla 2:** Distribución de los síntomas depresivos entre las mujeres residentes en el municipio de Vitória, ES, según los tipos de adversidades en la infancia – Vitória, ES, Brasil, 2022.

	N	%	IC del 95 %	Valor p*
**Negligencia emocional**				<0,001
No	174	21,3	18,6–24,2	
Sí	86	32,1		
**Negligencia física**				<0,001
No	139	19,0	16,3–22,0	
Sí	121	34,1	29,3–39,2	
**Abuso sexual**				<0,001
No	210	21,9	19,4–24,6	
Sí	50	39,1	31,0–47,8	
**Maltrato físico**				<0,001
No	94	17,6	14,6–21,0	
Sí	166	30,1	26,4–34,1	
**Abuso emocional**				<0,001
No	71	13,9	11,2–17,2	
Sí	189	32,9	29,1–36,8	
**Separación de los padres**				0,006
No	118	20,6	17,4–24,1	
Sí	142	27,7	24,0–31,8	
**Familiar encarcelado**				<0,001
No	219	22,0	19,5–24,7	
Sí	41	45,6	35,6–55,8	
**Familiar con problemas de salud mental**				0,001
No	193	21,8	19,2–24,7	
Sí	67	33,3	27,2–40,1	
**Abuso de alcohol por parte de familiares**				<0,001
No	155	20,3	17,6–23,3	
Sí	105	32,5	27,6–37,8	

N: frecuencia bruta.

%: frecuencia relativa.

IC95 %: Intervalo de confianza del 95 %.

valor p de la prueba de chi-cuadrado de Pearson.

Tras el análisis ajustado (Tabla 3), se observa que pertenecer al primer quintil de renta familiar se asocia con un aumento de 2,98 veces más probabilidades de presentar síntomas depresivos (IC del 95 %: 1,86–4,77) en comparación con las mujeres que pertenecen al grupo con mayor renta familiar. En cuanto a las adversidades en la infancia, las mujeres con antecedentes de negligencia física durante la infancia presentaron un aumento de 1,59 veces más probabilidades de presentar síntomas depresivos (IC del 95 %: 1,16–2,16) y aquellas que sufrieron abusos sexuales presentaron 1,57 veces más probabilidades (IC del 95 %: 1,03–2,39). Las mujeres con antecedentes de maltrato emocional durante la infancia presentaron 2,5 veces más probabilidades de presentar síntomas depresivos en la edad adulta (IC del 95 %: 1,82–3,44), al igual que las participantes que indicaron tener algún familiar encarcelado, que presentaron 1,90 veces más probabilidades de presentar el resultado objeto de estudio (IC del 95 %: 1,18–3,05).

**Tabla 3:** Análisis bruto y ajustado de los síntomas depresivos según los datos sociodemográficos, económicos y los tipos de adversidades en la infancia entre las mujeres residentes en el municipio de Vitória, ES, Brasil, 2022.

	OR	IC del 95 %	Valor p	OR ajustado	IC del 95 %	Valor p
**Grupo de edad**			0,222	–		
De 18 a 34 años	*Ref.*		–	–	
De 35 a 59 años	0,76	0,55–1,06		–	–	
≥60 años	0,76	0,52–1,12		–	–	
**Raza y color de piel**			0,412			–
Blanca	*Ref.*			–	–	
Negra	1,25	0,85–1,83		–	–	
Marrón y otras	0,97	0,71–1,33		–	–	
**Nivel educativo de la mujer**			0,035			0,250
De 0 a 4 años	1,94	1,12–3,35		1,60	0,92–2,80	
De 5 a 8 años	1,30	0,87–1,94		0,99	0,65–1,51	
De 9 a 11 años	*Ref.*			*Ref.*		
**Renta familiar**			<0,001			<0,001
1.º quintil (los más pobres)	2,98	1,86–4,77		2,98	1,86–4,77	
2.º quintil	2,25	1,39–3,65		2,25	1,39–3,65	
3.º quintil	1,30	0,78–2,15		1,30	0,78–2,15	
4.º quintil	1,64	1,00–2,71		1,64	1,00–2,71	
5.º quintil (los más ricos)	*Ref.*			*Ref.*		
**Negligencia emocional**			<0,001			0,107
No	*Ref.*			*Ref.*		
Sí	1,75	1,29–2,38		1,31	0,94–1,82	
Negligencia física			<0,001			0,003
No	*Ref.*			*Ref.*		
Sí	2,20	1,65–2,93		1,59	1,16–2,16	
**Abuso sexual**			<0,001			0,037
No	*Ref.*			*Ref.*		
Sí	2,28	1,55–3,36		1,57	1,03–2,39	
**Maltrato físico**			<0,001			0,375
No	*Ref.*			*Ref.*		
Sí	2,02	1,52–2,70		1,17	0,83–1,64	
**Abuso emocional**			<0,001			<0,001
No	*Ref.*			*Ref.*		
Sí	3,03	2,24–4,12		2,50	1,82–3,44	
**Separación de los padres**			0,006			0,786
No	*Ref.*			*Ref.*		
Sí	1,48	1,12–1,96		1,04	0,77–1,41	
**Familiar encarcelado**			<0,001			0,008
No	*Ref.*			*Ref.*		
Sí	2,99	1,91–4,61		1,90	1,18–3,05	
**Familiar con problemas de salud mental**			<0,001			0,106
No	*Ref.*			*Ref.*		
Sí	1,79	1,28–2,50		1,35	0,94–1,93	
**Abuso de alcohol por parte de familiares**			<0,001			0,813
No	*Ref.*			*Ref.*		
Sí	1,89	1,41–2,53		0,96	0,67–1,37	

IC95 %: Intervalo de confianza del 95 %.

Valor p de la prueba de chi-cuadrado de Pearson.

OR: odds ratio bruto (sin ajustes).

ORa: odds ratio ajustado.

## DISCUSIÓN

El presente estudio puso de manifiesto la importancia de identificar los factores de vulnerabilidad asociados a la salud mental de las mujeres, lo que contribuye a la planificación de acciones de atención de enfermería y a la implementación de estrategias de detección temprana de síntomas depresivos.

Se observa que el nivel educativo pierde significación estadística tras el ajuste de las variables en el modelo (p = 0,250). Este cambio sugiere una interrelación con otros factores documentados, como el ingreso. Según diversos estudios, la educación facilita la inserción en el mercado laboral y la autosuficiencia económica, lo cual, a su vez, reduce la exposición a factores estresantes crónicos y aumenta la capacidad de negociación y la autonomía de la mujer en sus contextos sociales, mitigando la probabilidad de que surjan síntomas depresivos y aumentando el acceso real a medios de protección contra los factores estresantes^(22,23)^.

En consonancia con esto, nuestros hallazgos revelaron que las mujeres que pertenecen al quintil de ingresos más bajos tenían 2,98 veces más probabilidades de presentar síntomas depresivos en comparación con las de ingresos más altos. Dicha asociación sugiere una mayor exposición a factores estresantes psicosociales crónicos entre las mujeres de menores ingresos —condiciones de vida desfavorables, menor apoyo social y acceso limitado a la educación y a los medicamentos—, factores que se reconocen como asociados al desarrollo de la depresión^(24)^.

En el contexto brasileño, caracterizado por una elevada desigualdad social y redes de protección frágiles, los ingresos se configuran como un indicador importante de privación material, lo que se refleja en las barreras que sobrecargan la capacidad de afrontamiento de las mujeres, aumentando la probabilidad de que se presenten síntomas depresivos. Este hallazgo también se observa en contextos internacionales, como lo muestra un estudio transversal que utilizó datos de la Encuesta Nacional de Salud y Nutrición de EE. UU., el cual señaló una asociación entre los ingresos y la depresión^(25)^.

En cuanto a las adversidades en la infancia, el abandono físico durante la infancia multiplicó por 1,59 las probabilidades de presentar síntomas depresivos (IC 95 %: 1,16–2,16). Este hallazgo concuerda con otro estudio que identificó el abuso psicológico y el abandono como factores asociados a la depresión^(26)^. A diferencia de las formas directas de violencia, la negligencia física priva al niño de cuidados básicos, generando sentimientos de soledad y aislamiento social. Esta carencia, que comienza en esta etapa, compromete el desarrollo de las habilidades sociales y la regulación emocional, lo que contribuye a la aparición del trastorno depresivo en la edad adulta^(27)^.

El abuso emocional se destacó como el predictor de mayor magnitud entre todas las adversidades investigadas (ORa: 2,50; IC95% 1,82–3,44).

Aunque es una de las formas más “silenciosas” de maltrato infantil, su fuerte asociación con la depresión en la vida adulta está relacionada con la vulnerabilidad al desarrollo de un estilo cognitivo negativo, lo que conlleva un mayor riesgo de depresión^(28,29)^. Esto se debe a la exposición, a menudo degradante, humillante y psicológicamente aterradora, al cuidador, lo que lleva a sentimientos de impotencia y a una baja autoestima en el niño. Esto culmina en un estilo cognitivo negativo que también se presenta en la etapa adulta, siendo un factor importante para la aparición de la depresión^(25)^. Además, desde la perspectiva de la Teoría del Apego, la falta de apoyo emocional^(30)^ y el establecimiento de un apego negativo predisponen a la internalización de los síntomas, lo que contribuye a que posteriormente se presenten cuadros depresivos en la vida^(31)^.

En cuanto al abuso sexual (ORa 1,57; IC 95 % 1,03–2,39), la evidencia sugiere que el abuso sexual puede llegar a “incorporarse biológicamente” al organismo a través de alteraciones en las vías de respuesta al estrés. La desregulación del eje hipotálamo-hipófisis-suprarrenal (HPA) y, en consecuencia, una mayor reactividad del cortisol, contribuyen directamente al desarrollo y mantenimiento de los síntomas depresivos^(32,33)^. Cabe destacar que esta asociación es particularmente sólida en casos de depresión atípica (hipersonia y aumento de peso) y está vinculada a sentimientos de encierro y habituación al dolor, lo que conlleva riesgos de conductas depresivas^(34)^.

El encarcelamiento de un familiar casi duplicó la probabilidad de presentar síntomas depresivos (IC 95 %: 1,18–3,05). Este hallazgo revela que el encarcelamiento de un miembro de la familia no es un evento aislado, sino que desencadena una serie de vulnerabilidades estructurales que afectan los aspectos sociales, económicos y de salud del niño. Además, la ausencia de los padres puede aumentar la probabilidad de una menor vigilancia y supervisión parental, lo que eleva el riesgo de conductas peligrosas y la posibilidad de que haya figuras sustitutas. Esta reconfiguración del núcleo familiar da lugar a otras formas de maltrato, incluido el abuso sexual, y, de este modo, crea un entorno de alta toxicidad emocional y mayores probabilidades de que surjan síntomas depresivos^(35)^.

Dichos cambios en el núcleo familiar, marcados por conflictos continuos y sentimientos de abandono, actúan como potentes factores estresantes crónicos que comprometen las habilidades relacionales y sociales del niño^(36)^. Además, esta situación estresante dentro de la familia genera una ruptura de la confianza y la aparición de inseguridad en el entorno doméstico, lo que también puede obstaculizar el desarrollo cognitivo, provocando daños psicológicos y dificultades escolares^(37)^.

Las evidencias aquí presentadas refuerzan la urgencia de contar con estrategias para la identificación de los EAI, ya que constituyen un grave problema de salud pública, con impactos negativos persistentes a lo largo de la vida. En este contexto, la enfermería desempeña un papel estratégico debido a su trabajo cercano a las comunidades y al acompañamiento de las mujeres a lo largo de su ciclo de vida.

El enfermero, al ser con frecuencia el profesional que establece el primer contacto con los usuarios de los servicios de salud, ocupa una posición privilegiada para la identificación temprana de vulnerabilidades. La calidad de la relación entre el paciente y el enfermero es un factor diferenciador fundamental, ya que crea un ambiente de confianza, estableciendo un vínculo que favorece la expresión de quejas, la revelación de historias, etc., lo que permite comprender que la depresión está relacionada con las experiencias vividas en la infancia. De este modo, el profesional puede planificar intervenciones, enfocarse en la promoción de la salud para la protección de la niña y en la derivación oportuna a la atención psicosocial. Se sugiere que estudios futuros investiguen estas interacciones específicas de las adversidades y si la privación material realmente amplifica los traumas relacionados.

Como limitación del estudio, podemos señalar la causalidad entre las variables estudiadas, así como el sesgo de selección, ya que solo se incluyeron mujeres que tenían o habían tenido una pareja íntima en los últimos 24 meses, lo que podría limitar la generalización de los hallazgos a la población femenina en general. Otro sesgo a destacar es el de memoria, ya que se utilizaron autorrelatos al plantear preguntas sobre la infancia a personas adultas y la participante podría haber olvidado cierta información. Además, existe un sesgo de información, ya que, al tratarse de un tema delicado, las mujeres entrevistadas podrían haber tenido dificultades para precisar la información proporcionada, lo que podría haber favorecido una posible subestimación de los datos. Sin embargo, para minimizar esta posibilidad, la investigación se llevó a cabo en un entorno privado, solo con la mujer y el investigador, lo que garantizó una mayor comodidad para la entrevistada.

## CONCLUSIÓN

Este estudio reafirma la asociación entre la exposición a adversidades en la infancia (abuso emocional y sexual, encarcelamiento de un familiar y negligencia física) y la privación socioeconómica como contextos de mayor vulnerabilidad a la sintomatología depresiva en mujeres adultas. Los hallazgos son de gran relevancia al proporcionar evidencia sólida basada en la población de una capital brasileña, un aspecto aún poco explorado en estudios nacionales.

Los resultados señalan la importancia de las estrategias de prevención e intervención temprana, así como del papel del personal de enfermería en este proceso, ya que con frecuencia es este profesional quien establece el primer contacto con la usuaria, ocupando una posición estratégica para la detección temprana de vulnerabilidades. Al establecer un enfoque orientado a la protección de la infancia, el fortalecimiento de los vínculos familiares y la reducción de las desigualdades sociales, junto con una perspectiva integral y sensible en los servicios de salud, se crea un paradigma de atención a la salud de la mujer y la niña, mitigando así los efectos nocivos de las situaciones vividas.

Por último, los hallazgos revelan cuán importante y necesaria es la inversión en políticas públicas intersectoriales para la protección de la infancia y la lucha contra las desigualdades económicas, a fin de minimizar los eventos adversos en esta importante etapa de la vida, ya que tienen un impacto negativo en la salud a lo largo de toda la vida.

## Data Availability

Todo el conjunto de datos que respalda los resultados de este estudio está disponible previa solicitud al autor correspondiente.
